# A data-driven machine learning algorithm to predict the effectiveness of inulin intervention against type II diabetes

**DOI:** 10.3389/fnut.2024.1520779

**Published:** 2025-01-07

**Authors:** Shuheng Yang, Ralf Weiskirchen, Wenjing Zheng, Xiangxu Hu, Aibiao Zou, Zhiguo Liu, Hualin Wang

**Affiliations:** ^1^School of Life Science and Technology, Wuhan Polytechnic University, Wuhan, China; ^2^Institute of Molecular Pathobiochemistry, Experimental Gene Therapy and Clinical Chemistry (IFMPEGKC), RWTH University Hospital, Aachen, Germany; ^3^Research Center of Medical Nutrition Therapy, Cross-strait Tsinghua Research Institute, Xiamen, China

**Keywords:** type 2 diabetes, inulin, machine-learning algorithm, treatment decision, XGBoost

## Abstract

**Introduction:**

The incidence of type 2 diabetes mellitus (T2DM) has increased in recent years. Alongside traditional pharmacological treatments, nutritional therapy has emerged as a crucial aspect of T2DM management. Inulin, a fructan-type soluble fiber that promotes the growth of probiotic species like *Bifidobacterium* and *Lactobacillus*, is commonly used in nutritional interventions for T2DM. However, it remains unclear which type of T2DM patients are suitable for inulin intervention. The aim of this study was to predict the effectiveness of inulin treatment for T2DM using a machine learning model.

**Methods:**

Original data were obtained from a previous study. After screening T2DM patients, feature election was conducted using LASSO regression, and a machine learning model was developed using XGBoost. The model’s performance was evaluated based on accuracy, specificity, positive predictive value, negative predictive value and further analyzed using receiver operating curves, calibration curves, and decision curves.

**Results:**

Out of the 758 T2DM patients included, 477 had their glycated hemoglobin (HbA1c) levels reduced to less than 6.5% after inulin intervention, resulting in an incidence rate of 62.93%. LASSO regression identified six key factors in patients prior to inulin treatment. The SHAP values for interpretation ranked the characteristic variables in descending order of importance: HbA1c, difference between fasting and 2 h-postprandial glucose levels, fasting blood glucose, high-density lipoprotein, age, and body mass index. The XGBoost prediction model demonstrated a training set accuracy of 0.819, specificity of 0.913, positive predictive value of 0.818, and negative predictive value of 0.820. The testing set showed an accuracy of 0.709, specificity of 0.909, positive predictive value of 0.705, and negative predictive value of 0.710.

**Conclusion:**

The XGBoost-SHAP framework for predicting the impact of inulin intervention in T2DM treatment proves to be effective. It allows for the comparison of prediction effect based on different features of an individual, assessment of prediction abilities for different individuals given their features, and establishes a connection between machine learning and nutritional intervention in T2DM treatment. This offers valuable insights for researchers in this field.

## 1 Introduction

The prevalence of type 2 diabetes mellitus (T2DM) around the world has reached a high level in recent decades. In China, the prevalence of T2DM among adults was 12.8%, and prediabetes was 35.2% (1). T2DM is a metabolic disease caused by both genetic and environmental factors, characterized by hyperglycemia, insulin resistance, and/or insulin secretion disorder. The Da Qing Diabetes Prevention Outcome Study has revealed that lifestyle intervention can effectively delay the onset of T2DM and reduce the incidence of diabetic complications in people with impaired glucose tolerance. This suggests that lifestyle intervention plays an important role in the prevention and treatment of T2DM (2). Nutritional therapy is an important component of lifestyle intervention (3, 4). The DiRECT study demonstrated that diet replacement and supervision lead to weight loss in obese T2DM patients, making the goal of remission in diabetes achievable (5). Remission in T2DM is defined as glycated hemoglobin (HbA1c) < 6.5% measured at least 3 months after cessation of glucose-lowering pharmacotherapy, which is a possible goal for T2DM treatment, including nutritional therapy (6). Dietary or energy restriction, low or very low carbohydrate diets, and bariatric surgery are potential strategies to achieve T2DM remission. In addition, scientific nutrition supplements to improve insulin sensitivity are another possible approach (5, 7, 8).

Insulin resistance is the central etiologic factor in T2DM, which is attributed to chronic systemic low-grade inflammation. Reversion of insulin sensitivity is essential for remission in T2DM (9). Chronic inflammation in tissues is at least partially induced by gut microbial dysbiosis, a typical feature in T2DM patients characterized by the accumulation of LPS-producing bacteria and other opportunistic pathogens, as well as weakening of the gut barrier (10, 11). A recent study indicated that two core competitive microbiomes in the gut play a central role in the development and intervention of diabetes, and high dietary fiber consumption leads to the restoration of gut microbial eubiosis (12). Dietary fiber can be utilized by gut microbiota as prebiotics to produce short chain fatty acids (SCFAs), particularly butyric acid. This supports the growth of colonic epithelial cells, repairs the gut barrier, and stimulates the secretion of GLP-1 in L cells, ultimately contributing to the remission of T2DM (13–16).

Inulin, a fructan-type soluble fiber, is a well-known prebiotic fiber that can support the proliferation of probiotic species *Bifidobacterium* and *Lactobacillus* and has been widely used in T2DM nutritional therapy. Several meta-analyses have summarized the protective effects of inulin against T2DM, showing that inulin supplementation can reduce fasting blood glucose (FBG) levels, HbA1c, and significantly improve insulin sensitivity in T2DM patients and prediabetic individuals (17–19). Our previous clinical trial revealed similar results: 12 weeks of consuming 20 g of inulin per day decreased HbA1c, FBG, 2-h postprandial glucose (2 h-PG) levels, as well as plasma total cholesterol and triglyceride levels (20). However, in reality, individuals with different ages, body mass indexes (BMIs), genders and time since diabetes diagnosis may have varying responses to inulin intervention. Accurately predicting the effectiveness will help identify T2DM patients suitable for inulin intervention. In the current study, we used our previous clinical data to develop a machine learning algorithm to predict the effectiveness of inulin intervention against T2DM. We hope our algorithm will be a valuable tool to support nutritional therapy in T2DM.

## 2 Design and methodology

### 2.1 Subject of the study

Data from 758 patients with T2DM who were treated at the People’s Hospital and Hospital of Traditional Chinese Medicine in Huangpi District, Wuhan City, was retrospectively collected. The data used in this study were obtained from our previous studies (in Chinese) (20, 21). In summary, both newly diagnosed and previously diagnosed T2DM patients received regular anti-diabetic medication for a 4-week observation period. Following this, they were instructed to take 20 g of inulin per day (provided by Wuhan Inulin Biotech Co.) in addition to their medical treatment for an additional 12 weeks. This study was approved by the Biomedical Ethics Committee of Wuhan Polytechnic University (BME-2022-1-13).

### 2.2 Characterization and screening of subjects

Basic information about the patients was collected, including gender, age, BMI, fasting blood glucose (FBG), two-hour postprandial glucose (2 h-PG), difference value between 2 h-PG and FBG (ΔPG, or DeltaPG), area under the curve (AUC) of blood glucose changes, HbA1c, total cholesterol (TC), triglycerides (TG), low-density lipoprotein (LDL), high-density lipoprotein (HDL), and HbA1c of patients before and after inulin intervention (Supplementary Files 1 and 2). Initially, the 758 patients were divided into a group that had an effect of receiving inulin intervention treatment and a group that did not have a significant effect of inulin intervention treatment based on their HbA1clevels below 6.5%. Secondly, the other indicators were analyzed individually using the “CBCgrps” package in R. The correlation between these variables was analyzed using the “corrplot” package. Subsequently, the “glmnet” package in R was utilized to analyze the correlation between these variables. The “glmnet” package in R was also used to screen the characteristic variables through LASSO regression (nfold = 20). The coefficients of the relevant independent variables were adjusted to zero to reduce the influence of multicollinearity on the regression results.

### 2.3 XGBoost machine learning for constructing predictive models

Before building the model, the dataset is divided into training and testing sets in a 7:3 ratio. The “XGBoost” package is then used to build the prediction model. Firstly, the feature variables are transformed into DMatrix format. Next, the train function is used to optimize the parameters by inserting the symbol package and outputting the best parameter configurations. Finally, the learning rate (eta) is set to 0.01, max_depth to 3, lambda to 1, subsample to 0.8, colsample_bytree to 0.8, and the number of iteration rounds to 100 to construct the XGBoost model. By leveraging the “caret” package in R, the model’s performance metrics, including accuracy, specificity, positive predictive value, and negative predictive value are computed. Additionally, receiver operating curves (ROCs), calibration curves, and decision curves are generated to assess and evaluate the model.

### 2.4 SHAP interpretation of the XGBoost model

The XGBoost model was interpreted using the “shapviz” package in R. Two samples were randomly selected to test their respective predicted SHAP values. Simultaneously, the total number of feature factors was analyzed, the contribution of each feature factor to the model’s prediction was interpreted, and the features were ranked in order of importance based on the average absolute value of the SHAP values.

### 2.5 Statistics analysis

All statistical analyses and data visualization were performed using R version 4.4.1. Continuous variables were presented as mean ± standard deviation or median (interquartile range (IQR)), while categorical variables were displayed as frequencies (percentages). The chi-square test was used for categorical variables, and independent samples *t*-tests or non-parametric tests were employed for continuous variables to assess differences between groups. The entire study was conducted using the software packages such as“CBCgrps,”“corrplot,”“glmnet,”“XGBoost,”“caret,” and“shapviz” for data management and visualization.

## 3 Results

### 3.1 Basic characteristics of patients

In the study, 758 patients with T2DM were analyzed. Out of these, 477 patients had their HbA1clevels reduced to less than 6.5% after receiving inulin intervention, indicating effective treatment. The remaining 281 patients showed insignificant treatment effects. The basic data of the 758 patients were categorized based on the outcome of inulin intervention, as presented in Table 1. The analysis revealed significant differences between the groups with positive and negative treatment effects in terms of age (*p* = 0.03), BMI (*p* = 0.009), FBG (*p* = 0.018), 2 h-PG (*p* < 0.001), DeltaPG (*p* < 0.001), AUC (*p* < 0.001), HbA1c (*p* < 0.001), and TG (*p* = 0.008).However, there were no significant variations in sex, TC, LDL, or HDL levels between the two groups (*p* > 0.05). The categorical variables in the table represent the number of cases (percentage), while the statistical methods utilized include the chi-square test, exact probability method, non-parametric test, and *t*-test for describing continuous variables such as median (percentile) or mean ± standard deviation.

**Table 1:** Basic information of the groups with poor and good effects of inulin intervention.

Variables	Total (*n* = 758)	Poor effect (*n* = 281)	Good effect (*n* = 477)	*p*
Sex, n (%)				0.535
Female	460 (61)	166 (59)	294 (62)	
Male	298 (39)	115 (41)	183 (38)	
Age (years)	60 (53, 67)	62 (55, 67)	60 (52, 66)	**0.03**
BMI (kg/m^2^)	23.3(21.9, 25.03)	23.63(22,25.76)	23.1(21.9,24.8)	**0.009**
FBG (mmol/L)	7.86 (6.8, 9.81)	8 (6.9, 10.3)	7.81 (6.8, 9.5)	**0.018**
2 h-PG (mmol/L)	13.2(10.5, 16.58)	14.9(11.8, 17.8)	12.3(10.1, 15.8)	**< 0.001**
ΔPG (mmol/L)	4.7(2.6, 7.8)	6.2 (3.35, 8.8)	4.3(2.3, 6.98)	**< 0.001**
AUC (mmol/L.min)	1,284(1068.5, 1529.5)	1,362(1,176, 1,620)	1,230(1,038, 1,470)	**< 0.001**
HbA1c (%)	7.2 (6.6, 8.3)	8 (7.2, 9.2)	6.8 (6.27, 7.7)	**< 0.001**
TC (mmol/L)	4.82 (3.97, 5.6)	4.84(3.96, 5.79)	4.8(3.98, 5.55)	0.297
TG (mmol/L)	1.32 (0.99, 1.9)	1.48(1.04, 2.12)	1.26(0.95, 1.84)	**0.008**
LDL (mmol/L)	2.72(2.14, 3.31)	2.81(2.16, 3.35)	2.68(2.12, 3.24)	0.14
HDL (mmol/L)	1.26 (1.07, 1.51)	1.26(1.05, 1.62)	1.26(1.08, 1.45)	0.594

^aBolding in the table indicates statistical significance of p < 0.05. bIndicators in the table are all recorded pre-intervention indicators. AUC, area under the curve, BMI, body mass index; FBG; FBG, fasting blood glucose; DeltaPG, difference value between 2 h-PG and; HbA1c, glycated hemoglobin, HDL, high density lipoprotein; LDL, low density lipoprotein; TC, total cholesterol; TG, total glucose.^

### 3.2 One-way analysis of variance and LASSO regression for feature selection

**Figure 1:**
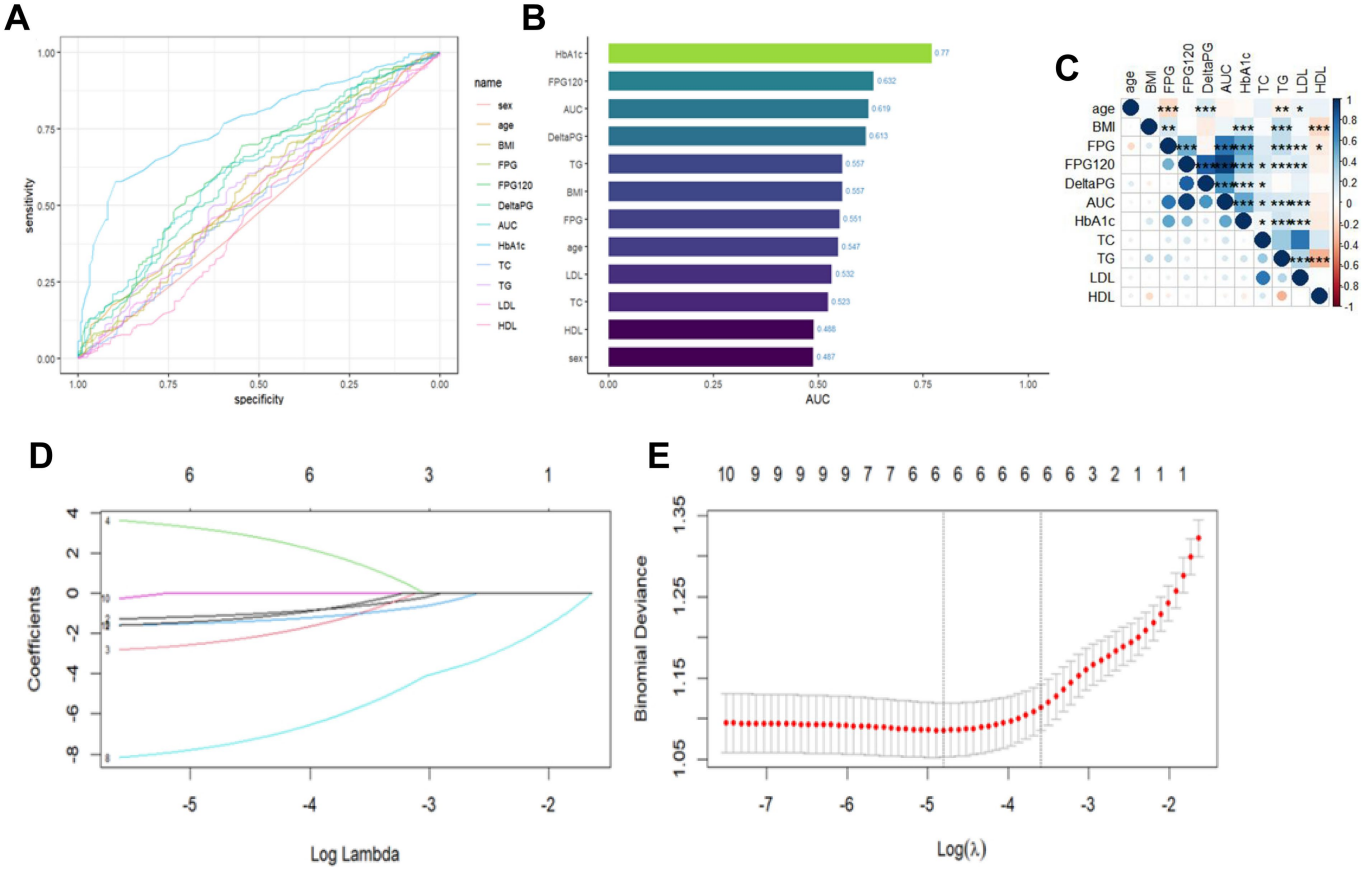
Analysis of individual variables and LASSO regression outcomes. **(A)** Plot of individual variables. **(B)** Plot of AUC values of individual variables. **(C)** Correlation heat map of continuous variables, where color depth represents correlation coefficient (r value), *p < 0.05; **p < 0.01; ***p < 0.001. **(D)** Plot of LASSO coefficient paths. **(E)** LASSO regression analysis of crossover curves.

The results of the case-by-case analysis for the included variables are depicted in Figures 1A,B. Additionally, the correlation heatmap revealed correlations among these variables (Figure 1C). To prevent covariance issues from affecting the construction of the prediction model later on, Lasso regression was used to select features from the included variables (Figure 1D). To ensure a good fit for the model, the *λ* corresponding to the minimum mean square error (lambda.min) was chosen after cross-validation (Figure 1E). Ultimately, we identified the four most predictive characteristic variables, which include “age,” “BMI,” “FBG,” “DeltaPG,”"HbA1c,” and “HDL.”

### 3.3 Construction of XGBoost prediction model and performance evaluation

**Figure 2:**
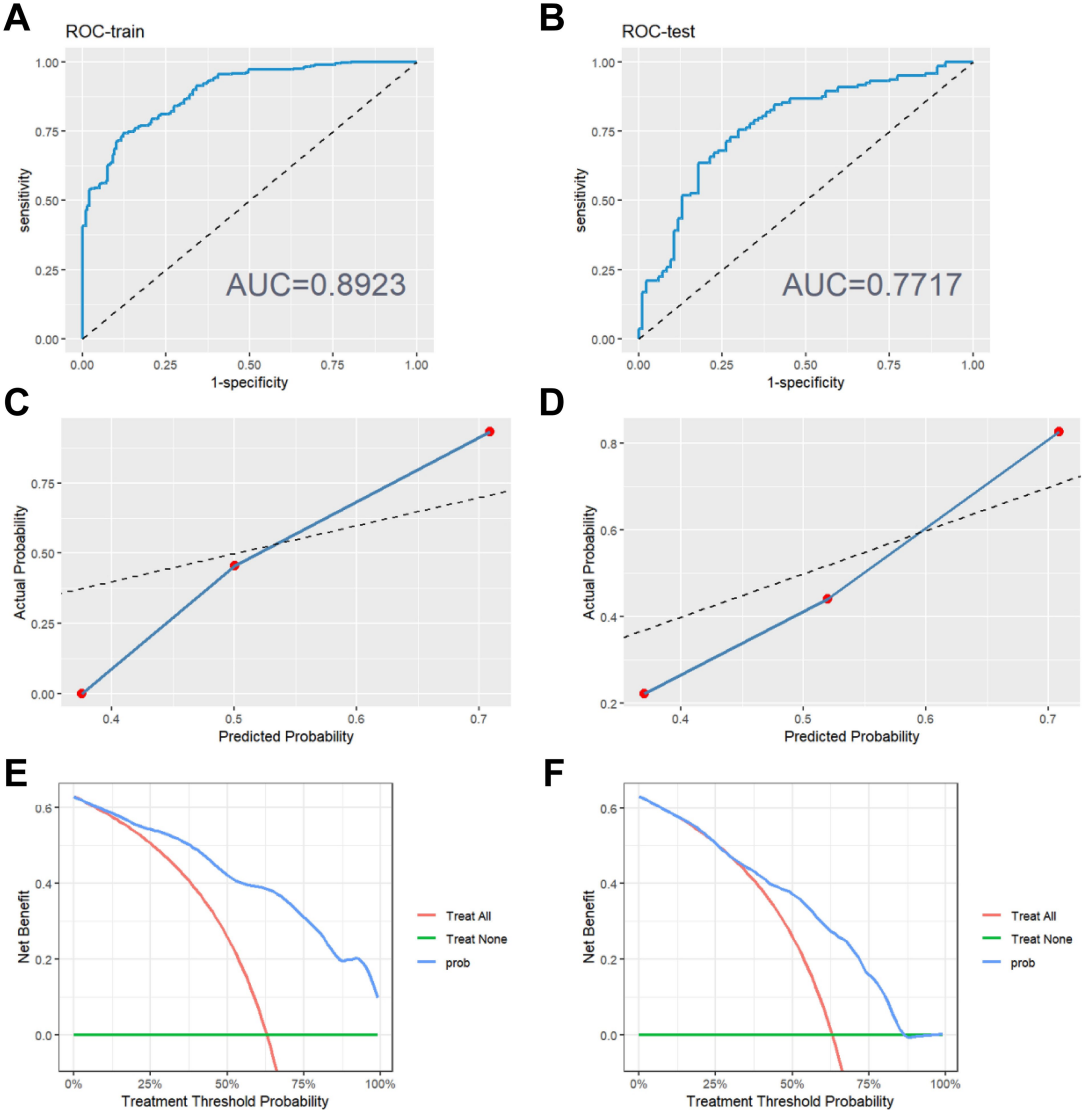
Evaluation of model performance. **(A)** ROC curve on the training set. **(B)** ROC curve on the testing set. **(C)** Calibration curve on the training set. **(D)** Calibration curve on the testing set. **(E)** Decision making curve on the training set. **(F)** Decision making curve on the testing set.

The performance evaluation metrics of the model are presented in Table 2. The XGBoost prediction model shows an accuracy of 0.819, specificity of 0.913, positive predictive value of 0.8181, and negative predictive value of 0.820 in the training set. The testing set demonstrates an accuracy of 0.709, specificity of 0.909, positive predictive value of 0.705, and negative predictive value of 0.710.Both accuracy and specificity indicate how well the predicted results align with the true values. Positive predictive value (PPV) signifies the likelihood that a person with a positive diagnostic test result is indeed a patient. Conversely, negative predictive value (NPV) indicates the probability that a person with a negative diagnostic test result is not a patient. The XGBoost model displays higher accuracy, specificity, positive predictive value, and negative predictive value in the training set. Although the accuracy, specificity, positive predictive value and negative predictive value of the testing set are also high, they are lower than those of the training set. This demonstrates that our XGBoost prediction model can to some extent reflect the therapeutic effect of the inulin intervention. Furthermore, we analyzed the ROC curves of the training and testing sets, with the area under the ROC curve reaching 0.892 for the training set (Figure 2A) and 0.771 for the testing set (Figure 2B). The higher area under the curve in both sets indicates strong model fidelity. The results of the calibration curves (Figures 2C,D) reveal that the predicted and actual value curves of the training and testing sets align relatively well, suggesting that the XGBoost model holds predictive significance. The decision curves for the training set (Figure 2E) outperform those of the validation set (Figure 2F), indicating that the model offers a favorable net benefit for clinical decision-making across most threshold probabilities.

**Table 2:** Performance evaluation of XGBoost model in training and test sets.

	Training set	Testing set
Accuracy	0.819 (0.783–0.851)	0.709 (0.623–0.747)
Specificity	0.913 (0.883–0.943)	0.909 (0.862–0.956)
PPV	0.818 (0.758–0.878)	0.705 (0.570–0.839)
NPV	0.820 (0.781–0.859)	0.710 (0.645–0.776)

The contents of the table in parentheses indicate 95 per cent confidence intervals in parentheses. NPV, negative predictive value; PPV, positive predictive value.

### 3.4 SHAP values to explain the feature factors in the XGBoost model

**Figure 3:**
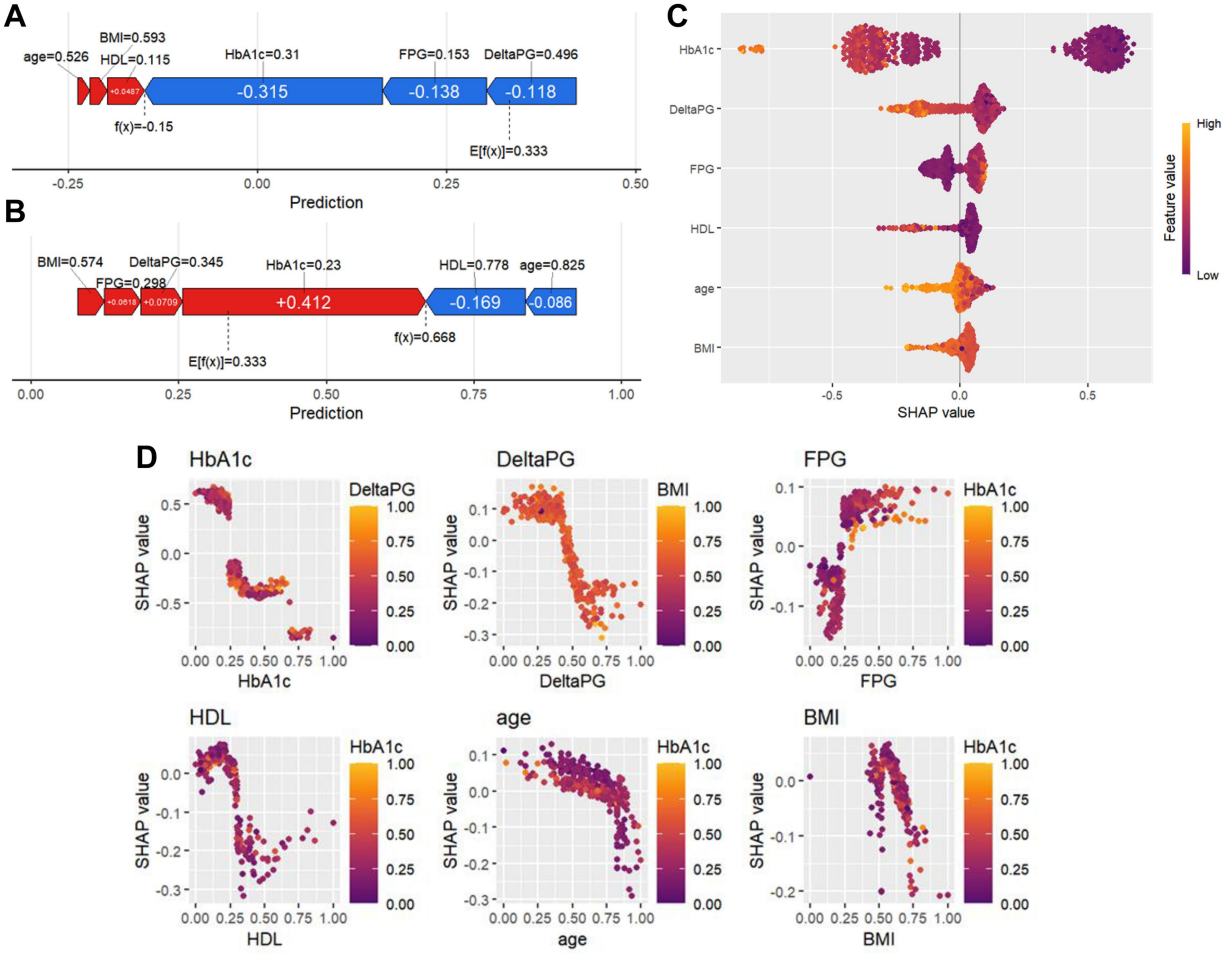
SHAP force diagrams and variable importance analysis for individual patients. **(A,B)** SHAP force diagrams for two random patients: the colors indicate the contribution of each feature, with blue indicating a negative impact on the prediction (left arrow, decrease in SHAP value) and red indicating a positive impact on the prediction (right arrow, increase in SHAP value). The length of the color bar indicates the strength of the contribution, and E[f (x)] denotes the SHAP reference value, which is the average of the model’s predictions. f(x) denotes the individual’s SHAP value. **(C)** Importance plot of SHAP variables, with included features sorted from highest to lowest mean absolute value of SHAP. **(D)** SHAP variable dependency plot.

The SHAP value allows for a random inspection of the predictive value of any single sample in the model and the influence of its characteristics on the predictive value. Therefore, we randomly selected two patients and used the SHAP force diagram to explain the contribution of their characteristic factors in the model. From left to right, the values of each characteristic factor are shown in order. The red bar demonstrates the positive potential value of the patient, while the blue bar indicates the negative potential value. E[f(x)] denotes the output mean of the XGBoost model, and f(x) represents the SHAP value of a single sample. The SHAP force diagram of the 1^st^ patient is shown in Figure 3A. We found that the effective mean value of the XGBoost model for the inulin intervention treatment is 0.333. The 1^st^ patient’s SHAP value reaches −0.150, which is less than the effective value, indicating that inulin intervention treatment was not effective for this patient. Similarly, the SHAP value of the 2nd patient was 0.668, as shown in Figure 3B, which is higher than the predicted mean value of 0.331, indicating an improvement in the patient’s T2DM with the inulin intervention treatment. In addition to visualizing the SHAP values of individual samples, it is also possible to visualize the characteristic factors (Figure 3C), where the horizontal coordinate is the SHAP value. The more yellow the color, the larger the value of the feature and its contribution to the model. Conversely, the more purple the color, the smaller the value. Therefore, we determined that the features affecting inulin intervention for the treatment of patients with T2DMare, in descending order, “HbA1c,” “FBG,” “DeltaPG,” “HDL,” “age,” and “BMI.” The SHAP values also reflect the degree of dependence of these features on the XGBoost model separately (Figure 3D). Each point in the figure represents a sample, with the horizontal axis being the eigenvalue of each feature, and the corresponding vertical axis being its SHAP value.

## 4 Discussion

Nutritional therapy has been widely used in the intervention of T2DM, and the supplementation of dietary fiber has been identified as an efficient way to improve T2DM nutritional therapy (4, 22). High intake of soluble dietary fiber has shown benefits for individuals with T2DM, not only in improving glycemic control but also in ameliorating hypercholesterolemia in these patients (23). The effects of different dietary fibers on diabetes vary, and while inulin may not be the most effective fiber, it is one of the most widely used and researched fibers that offers benefits for both glycemic and lipid control (24). In addition, inulin supplementation during pregnancy has been shown to improve glucose tolerance in offspring through SCFA fermentation (25, 26). Previous clinical trials have also demonstrated that inulin supplementation can reduce fasting and postprandial blood glucose levels, as well as improve hypertriglyceridemia and hypercholesterolemia in patients with T2DM or prediabetes (20, 21, 27). However, recent studies have suggested that high doses of inulin consumption may disrupt bile acid metabolism, leading to type 2 inflammation or even cholestatic liver cancer if dysbiosis of the gut microbiome exists (28–31). This indicates that the application of inulin should be approached with caution and precision. Identifying T2DM patients who would benefit from inulin intervention is crucial for precise nutritional therapy in the treatment of T2DM.

In the present study, we used our previous clinical data to create a machine-learning algorithm based on XGBoost. XGBoost is a modified tree boosting machine learning technique commonly used in predicting disease outcomes with clinical bigdata (32). Our algorithm shows that age, FBG, ΔPG, HbA1c, BMI, and HDL cholesterol levels are the most significant indicators for predicting the effects of inulin intervention (Figure 3), while 2 h-PG, AUC of blood glucose changes, TC, TG and LDL are not.

Three blood glucose-related factors, FBG, HbA1c, and ΔPG, were identified as predictive indicators (Figure 3). A clinical study analyzed the relationship between ΔPG and the characteristics of insulin resistance and insulin secretion in Chinese T2DM patients. It revealed that ΔPG was closely related to glucose effectiveness and insulin secretion phase change, suggesting that ΔPG is an important index to assess the severity of T2DM development (33).

Aging is an important risk factor for T2DM, and it is more challenging for elderly patients to recover from insulin resistance (34, 35). Moreover, an animal study has shown that the capacity for SCFAs fermentation was reduced in old mice compared to middle-aged mice, which may explain why patient age was identified as an effective indicator for inulin intervention (36). However, clinical studies have found that inulin-type fructans provide some benefits in the elderly. Although the effects may not be as strong as in younger individuals, elderly patients should still consume inulin for nutritional therapy (37).

In most cases, obesity is correlated with T2DM and dyslipidemia. This is because chronic inflammation and insulin resistance can lead to both obesity and T2DM, with obese T2DM patients benefiting more from nutritional therapy (5, 38). The effects of inulin administration on bodyweight have been inconsistent, with initial gut microbiota possibly playing a role in the feedback of inulin against obesity. However, the effects of inulin on insulin sensitivity are clear, as inulin supplements can significantly improve insulin resistance in individuals who are obese or overweight (17, 39, 40). BMI was identified as a predictive indicator in the model (Figure 3). HDL was another indicator (Figure 3), and the relationship between HDL and inulin intervention is more complex, T2DM patients may have abnormal HDL levels, both in terms of HDL-cholesterol level changes and the composition of HDL particles (41). Our previous study and other clinical studies have found that inulin can help elevate HDL-cholesterol levels in T2DM patients (18, 20).

The limitations of the current study include the small sample size and the fact that only blood glucose and lipid levels of patients were used for the prediction model. Critical information about the patients was also missing, such as the analysis of their gut microbiome before and after inulin supplementation, their medication status, and inflammatory indicators like C-reactive protein and TNF-*α* levels. Including this information could help further explore the personalized effects of inulin intervention against T2DM and minimize uncertainties in the usage of inulin supplements.

In conclusion, we constructed an algorithm based on XGBoost and found that the features affecting inulin intervention for T2DM were FBG, ΔPG, HbA1c, BMI index and HDL-c level. We hope our algorithm can help promote precise nutritional therapy for T2DM patients.

## Data Availability

The original contributions presented in the study are included in the article/Supplementary material, further inquiries can be directed to the corresponding authors.
