# Supplementary material for: A data-driven machine learning algorithm to predict the effectiveness of inulin intervention against type II diabetes
Source: Front Nutr. 2025 Jan 7;11:1520779. doi: 10.3389/fnut.2024.1520779 (PMC11747270; doi:10.3389/fnut.2024.1520779)
Supplement: Supplementary file 1 [file Data_Sheet_1.pdf]

DOI:10.16658/j.cnki.1672-4062.2018.11.012

益生元对 2 型糖尿病患者血糖控制和血脂代谢效果的影响

王盼[1],邹爱标[1,2],徐亥[3],游海军[1],孔苹苹[1],汪荣[1],彭淑珍[3],游静文[3],张炎生[4],夏卉琴[4]

1.武汉英纽林生物科技有限公司,湖北武汉　430000;2.清华大学生命科学与医学研究院-英纽林肠道菌群与代谢健康研究中心,北京　100084;3.黄陂区人民医院内分泌科、健康管理中心,湖北武汉　430300;4.武汉市黄陂中医院内分泌科、健康管理中心,湖北武汉　430300

[摘要] **目的** 验证菊粉型益生元对 2 型糖尿病患者血糖控制和血脂代谢的影响。**方法** 选取 2016 年 9 月—2017 年 9 月在黄陂区人民医院和中医院就诊的 2 型糖尿病患者 588 例,平均年龄为(56.19±9.83)岁,每例患者经过 2 个试验周期,观察期(为期 4 周)给予单纯药物治疗,营养干预期(为期 12 周)在药物治疗基础上,接受菊粉益生元营养强化干预。**结果** 经过益生元营养强化干预后,主要疗效指标 HbA1c 和次要指标 FBG、2 hPG、CHOL、TRIG、LDL、BMI、BP 与试验前和观察期第 4 周时比较显著降低,差异有统计学意义(P<0.05),HDL 对比试验前差异无统计学意义,但对比观察期第 4 周,HDL 升高,差异有统计学意义(P<0.01)。**结论** 菊粉型益生元干预有利于 2 型糖尿病患者的体重、血压、血糖、血脂的控制,营养强化干预比单纯的药物治疗方式效果更佳。

[关键词] 菊粉;益生元;2 型糖尿病;血糖;血脂

[中图分类号] R587.1;R151.3　　[文献标识码] A　　[文章编号] 1672-4062(2018)06(a)-0012-04

Effect of Prebiotic on the Blood Glucose Control and Lipid Metabolism of Patients with Type 2 Diabetes

WANG Pan[1], ZOU Ai-biao[1,2], XU Hai[3], YOU Hai-jun[1], KONG Ping-ping[1], WANG Rong[1], PENG Shu-zhen[3], YOU Jing-wen[3], ZHANG Yan-sheng[4], XIA Hui-qin[4]

1.Wuhan English Biotechnology Co, Ltd, Wuhan, Hubei Province, 430000 China;2.Institute of Life Science and Medicine of Tsinghua University—Enteric Flora and Metabolic Health Center, Beijing, 100084 China;3.Department of Endocrinology & Health Management Center, Huangpi District People's Hospital, Wuhan, Hubei Province,430300 China;4.Department of Endocrinology & Health Management Center, Wuhan Huangpi Hospital of TCM, Wuhan, Hubei Province, 430300 China

[Abstract] Objective To verify the effect of prebiotic on the blood glucose control and lipid metabolism of patients with type 2 diabetes. **Methods** 588 cases of patients with type 2 diabetes diagnosed in our hospital from September 2016 to September 2017 were selected,(56.19±9.83)years old on average, after two test cycles, during the observation period (four weeks), the simple drugs were given, during the nutrition intervention period (12 weeks), the intensive intervention of inulin prebiotic were given on the basis of the drugs. **Results** After the intensive intervention of prebiotic, the major curative effect indicator HbA1c and second indicators FBG, 2 hPG,CHOL, TRIG, LDL, BMI, BP obviously decreased compared with those before test and during the observation period, and the differences were statistically significant (P<0.05), and the difference in the HDL before and after test was statistically significant, but the HDL increased compared with that in the fourth week during the observation period, and the difference was statistically significant(P<0.01). **Conclusion** The inulin prebiotic intervention is conductive to controlling the weight, blood pressure, blood glucose and blood liquid of patients with type 2 diabetes, and the nutrition intensive intervention has a better effect than the simple drug treatment method.

[Key words] Inulin; Prebiotic; Type 2 diabetes; Blood glucose; Blood liquid

随着生活方式和饮食结构的改变,糖尿病在全球及中国的发病率迅速上升,已经成为严重的公共卫生问题[1],然而 2 型糖尿病患者血糖控制的现状却仍不理想[2]。医学营养治疗是糖尿病患者应该长期坚持的基础性治疗措施,根据糖尿病患者的病情给予合理的营养指导能够有效的控制糖尿病患者体重,提高胰岛素对肝脏、肌肉及脂肪组织等组织器官的敏感程度,增强胰岛素作

[作者简介] 王盼(1991-),女,湖北黄石人,硕士,研究方向:肠道菌群与代谢健康。

[通讯作者] 邹爱标(1970-),男,北京人,硕士,高级工程师,研究方向:肠道菌群与代谢健康、他汀类药物研发、慢性病医学营养治疗,E-mail:zouaibiao@inuling.com。

(C)1994-2019 China Academic Journal Electronic Publishing House. All rights reserved.　http://www.cnki.net

用能力，改善患者体内高血糖水平[3]。近年来学者们发现菊粉这种功能性食品可代替药物用于预防糖尿病，成为糖尿病医学营养治疗的有效措施。菊粉是一种水溶性膳食纤维，因其具有不被人体所消化吸收，但可以被肠道微生物利用发酵，选择性的促进双歧杆菌、乳酸杆菌等有益菌群的生长和代谢能力，因此又是典型的益生元。国内外已有很多研究证实菊粉益生元在糖尿病治疗中有很好的辅助治疗效果，有利于促进对血糖和血脂的控制作用[4-7]，但仍存在补充剂量较小、观察时间较短及观察样本数量较少的问题，在临床上应用较少，还需大样本的人群研究进一步验证。因此，该研究通过对2016年9月—2017年9月收治的588例2型糖尿病患者在单纯药物治疗基础上增加菊粉益生元营养强化治疗，观察16周的血糖、血脂控制情况，验证其临床应用效果，预防和延缓糖尿病及其并发症的发生和发展。

1 资料与方法

1.1 研究对象

选取在武汉市黄陂区人民医院和中医院就诊的2型糖尿病患者。纳入标准：①符合WHO的糖尿病诊断标准(1999)为2型糖尿病患者；②年龄≥18岁且≤80岁，性别不限；③自愿参加并签署知情同意。排除标准：①有严重的糖尿病并发症、严重肝肾功能不全、严重血液系统疾病、精神障碍及其他内分泌系统疾病者；②有改变胃肠道正常结构的手术史者；③存在应激性状态或有继发性血糖升高因素者（如服用糖皮质激素者）；④妊娠或哺乳期的妇女，计划妊娠或研究期间不愿避孕的妇女；⑤药物或其它毒品滥用者；⑥有药物过敏者；⑦3个月内参加过其他临床试验者。

1.2 治疗方法

试验过程分为2个周期，共16周。第1周期观察期为期4周，给予单纯药物治疗。第2周期为营养干预期为期12周，在药物治疗基础上，接受益生元(纽畅菊粉益生元,)营养强化干预，2次/d，10 g/次，餐前用温开水冲服。采用电话随访或门诊约谈的方式对患者进行全程监督和指导。

1.3 观察指标

体格检查包括身高、体重、血压，并计算体质指数[BMI=体重(kg)/身高(m)^2]。血糖检测指标包括糖化血红蛋白、空腹血糖、餐后2 h血糖。血脂检测指标包含总胆固醇、甘油三酯、高密度脂蛋白、低密度脂蛋白。糖化血红蛋白为主要疗效指标，其他指标为次要疗效指标。

1.4 统计方法

采用SAS 9.4统计学软件进行统计分析。计量资料以($\bar{x}\pm s$)表示，采用t检验、配对t检验、秩和检验、配对秩和检验、中位数检验等方法；计数资料以(%)表示，采用χ^2检验、Fisher精确检验等；等级资料采用Ridit分析，CMH法；全局评价指标、主要疗效指标同时进行PP分析和FAS分析；统计检验采用双侧的差异性检验，$P<0.05$为差异有统计学意义。

2 结果

2.1 一般资料结果

选取2型糖尿病患者共588例，其中男性225例，女性363例，年龄在21~80岁之间，平均年龄为(56.19±9.83)岁。病程在0.1~30年之间，平均病程(6.2±5.36)年。患者均无过敏史。

BMI是目前国际上常用的衡量人体胖瘦程度以及是否健康的一个标准，中国人BMI正常参考标准为18.5~23.9 kg/m^2，由表1可看出2型糖尿病患者的BMI稍偏高，说明大部分2型糖尿病患者体型超重，而经过益生元营养强化干预后第16周BMI对比试验前和观察期第4周分别下降了0.74%和1.11%，差异有统计学意义，这说明菊粉型益生元有利于2型糖尿病患者控制体重。

治疗16周后收缩压与试验前和4周时比较分别下降了2.19%和1.13%，差异有统计学意义($P<0.01$)，试验前和观察期的舒张压差异无统计学意义($P>0.05$)，但是服用12周菊粉益生元后舒张压下降，且差异有统计学意义($P<0.01$)，说明菊粉益生元对2型糖尿病患者的血压情况有一定改善作用。

表1 BMI和血压结果比较($\bar{x}\pm s$)

时间	BMI(kg/m^2)	收缩压(mmHg)	舒张压(mmHg)
试验前(第1天)	24.18±2.73	128.60±14.05	80.11±8.51
观察期(第4周)	(24.27±2.70)**	(127.42±11.34)*	80.23±8.05
营养干预后(第16周)	(24.00±2.74)**△△	(125.98±11.28)**△△	(78.57±7.37)**△△

注：**表示与试验前相比，$P<0.01$；*表示与试验前相比，$P<0.05$；△△表示与观察期第4周相比，$P<0.01$。

2.2 血糖结果比较

血糖指标结果如表2所示，结果表明，主要疗效指标糖化血红蛋白和次要指标空腹及餐后2 h血糖在试验前后均差异有统计学意义($P<0.01$)，血糖指标显著降低。与试验前相比，观察期4周(单纯药物治疗方式)糖化血红蛋白、空腹血糖和餐后2 h血糖分别下降了3.36%、4.62%、7.15%，益生元营养强化干预后的糖化血红蛋白、空腹血糖、餐后2 h血糖比观察期又分别下降了12.24%、17.86%、19.44%，可看出，利用菊粉益生元营养强化干预方法比单纯的使用药物治疗血糖控制效果更好。

(C)1994-2019 China Academic Journal Electronic Publishing House. All rights reserved. http://www.cnki.net

表 3 血脂指标情况[($\bar{x}\pm s$),mmol/L]

时间	CHOL	TRIG	HDL	LDL
试验前(第 1 天)	4.84±1.30	1.79±1.73	1.34±0.44	2.68±0.87
观察期(第 4 周)	(4.67±1.12)**	(1.65±1.32)**	1.30±0.40	(2.53±0.81)**
营养干预后(第 16 周)	(4.31±1.10)**△△	(1.43±0.89)**△△	(1.38±0.44)△△	(2.41±0.83)**△△

注:** 表示与试验前相比,$P<0.01$;△△表示与观察期第 4 周相比,$P<0.01$。

表 2 血糖指标情况($\bar{x}\pm s$)

时间	HbA1c(%)	FPG(mmol/L)	2 hPG(mmol/L)
试验前(第 1 天)	7.44±1.56	9.10±2.93	13.00±4.70
观察期(第 4 周)	(7.19±1.27)**	(8.68±2.46)**	(12.09±4.19)**
营养干预后(第 16 周)	(6.31±0.97**△△	(7.13±1.57)**△△	(9.75±2.13)**△△

注:** 表示与试验前相比 $P<0.01$,△△表示与观察期第 4 周相比 $P<0.01$。

2.3 血脂结果比较

血脂指标结果如表 3 所示,与试验前相比,观察期 4 周(单纯药物治疗)后总胆固醇、甘油三酯、低密度脂蛋白分别下降了 3.51%、7.82%、5.60%,再经过益生元营养强化干预后下降趋势更明显,与观察期比较,总胆固醇、甘油三酯、低密度脂蛋白又分别下降了 7.7%、13.33%、4.74%,差异有统计学意义($P<0.01$)。虽然高密度脂蛋白与试验前比较差异无统计学意义($P>0.05$),但是相比观察期升高了 6.15%,差异有统计学意义($P<0.01$)。由此可看出利用菊粉益生元营养强化干预方法比单纯的使用药物治疗控制血脂效果更理想。

3 讨论

该研究中干预对象对菊粉益生元的耐受性较好,未出现明显的不良反应,显示其具有良好的安全性,与国内外的报道一致[8-9]。

糖尿病的发生与遗传因素、生活方式、环境等因素密切相关,因此糖尿病的治疗不仅要求专业的医疗知识和技术,也对生活方式和饮食结构的改变有着非常强的依赖性[10]。医学营养治疗是糖尿病患者应该长期坚持的、基础性的治疗措施。孙中明等[11]研究显示,对 T2DM 患者进行饮食干预可以有效改善患者的血糖水平。对 2 型糖尿病患者采用药物联合饮食、运动强化治疗的方式不仅可以更好的降低血糖,还能防止糖尿病并发症发生[12]。

该研究每天给患者补充 20 g 菊粉益生元,持续营养强化 12 周后,糖化血红蛋白、空腹血糖、餐后 2 h 血糖相比试验前和观察期均有明显降低,血脂代谢明显改善,患者的体重也得到控制。研究表明,益生元被肠道菌群利用的酵解产物短链脂肪酸(如丙酸、乙酸、丁酸等)可以通过结合游离脂肪酸受体来下调炎症因子和上调 GLP-1 等胃肠激素分泌,参与血糖平衡调节,改善代谢性炎症和胰岛素抵抗[13]。此外,丙酸影响肝脏的脂肪生成和糖异生,而菊粉还可以吸附肠道中阴离子和胆汁酸从而有效降低血脂和胆固醇[14]。

该研究患者血糖指标的降低可能还与菊粉中膳食纤维改善胰岛素敏感性有关。胰岛素抵抗是 2 型糖尿病发病机制中的一个重要因素,低胰岛素敏感性是代谢综合征的基础[15]。有研究证实膳食纤维摄入量的增加能改善胰岛素的敏感性[16]。这为 2 型糖尿病的病因治疗提供了可能。因此在糖尿病营养治疗方案中强化膳食纤维和益生元在糖尿病治疗中的作用,通过长期的医学营养治疗,力求实现部分糖尿病患者对胰岛素的敏感性恢复至正常水平,以期达到取消药物治疗的目的[17]。另外,该研究在试验方法上仍有一定局限,未进行对照组试验,因此可能存在不能明确区分益生元和药物治疗效果的问题,还需补充更多更严谨的试验设计,以确证菊粉益生元对 2 型糖尿病患者的血糖和血脂水平的作用。

[参考文献]

[1] 徐瑜,毕宇芳,王卫庆,等.中国成人糖尿病流行与控制现状—2010 年中国慢病监测暨糖尿病专题调查报告解读[J].中华内分泌代谢杂志,2014,30(3):184-186.

[2] 孙飞,王丽,高彬,等.2 型糖尿病患者血糖控制情况调查分析[J].陕西医学杂志,2014,43(5):622-625.

[3] 贺婧,郭延玲.医学营养治疗联合低聚果糖干预对糖尿病患者血糖控制效果观察[J].广西医科大学学报,2018,35(3):402-405.

[4] Dehghan P, Pourghassem Gargari B, Asgharijafarabadi M. Effects of high performance inulinsupplementation on glycemic status and lipid profile in women with type 2 diabetes:arandomized, placebo-controlled clinical trial[J].Health Promot Perspect,2013,3(1):55-63.

[5] Jackson KG,Taylor GR,Clohessy AM,et al.The effect of the daily intake of inulin on fastinglipid,insulin and glucose concentrations in middle-aged men and women[J].Br J Nutr,1999,82(1):23-30.

[6] Russo F,Riezzo G,Chiloiro M,et al.Metabolic effects of a diet with inulin-enriched pasta inhealthy young volunteers[J].Curr Pharm Des,2010,16(7):825-831.

[7] F Liu,M Prabhakar,J Ju,et al.Effect of inulin-type fructans on blood lipid profile and glucoselevel: a systematic review and meta-analysis of randomized controlled trials[J].Eur J Clin Nutr,2017,71(1):9-20.

(下转第 95 页)

(C)1994-2019 China Academic Journal Electronic Publishing House. All rights reserved. http://www.cnki.net

囊切除术治疗过程更为复杂。且患者行腹腔镜胆囊切除术后,由于手术的应激反应以及血糖因素影响的双重因素,使得患者术后发生并发症的概率要远高于普通胆结石患者[5-6]。鉴于此,寻找患者可以接受手术治疗的最佳时机,同时在患者术后给予必要的护理干预,是提高治疗效果,降低术后并发症发生率的关键。

该院对胆结石伴糖尿病患者腹腔镜胆囊切除术围手术期给予了个体化护理干预,为患者手术治疗全程实施了个性化、专业化、综合化的护理。首先通过术前基本护理干预,调整了患者手术治疗心态,同时通过饮食和药物对患者血糖水平进行了有效的控制, 通过综合评估,为患者进行手术治疗选择了最佳的治疗时机。此外,对患者术中以及术后也给予了有效的护理干预措施。通过对各种导管的维护和管理,以及对患者注射胰岛素等方式,强化了患者术后预后过程,降低了可能导致患者出现感染的各种因素的出现,也就有效控制了患者术后的并发症发生率。

综上所述,对胆结石伴糖尿病患者采用腹腔镜胆囊切除术进行治疗的围手术期,为患者给予个体化护理干预,能够提升服务治疗,使患者满意度得到极大提高,同时还可以有效改善患者的焦虑、抑郁等负面情绪。

[参考文献]

[1] 王英.个体化围手术期护理对胆结石伴糖尿病患者腹腔镜胆囊切除术治疗效果的影响[J].河南医学研究,2017,24(17):3249-3250.

[2] 王海燕.临床护理路径在糖尿病患者行腹腔镜胆囊切除术中的应用效果评价[J].中华现代护理杂志,2013,15(33):4133-4135.

[3] 常凤华.腹腔镜胆囊切除术治疗胆结石合并糖尿病患者围术期护理效果的临床分析[J].糖尿病新世界,2015,13(13):122-123.

[4] 李秀荣.糖尿病合并胆结石患者行腹腔镜下胆囊切除术的围手术期护理[J].现代诊断与治疗,2017,54(9):1743-1745.

[5] 黄新波.胆结石合并糖尿病患者的护理干预研究[J].医药前沿,2018,15(4):286.

[6] 侯舒依.个体化护理对于胆结石手术护理患者的临床效果[J].养生保健指南,2018,52(6):80.

(收稿日期:2018-03-05)

(上接第 14 页)

[8] 刘鹏举,马方,李明,等.菊粉和金玉兰对 2 型糖尿病患者血糖控制和血脂代谢的影响[J].协和医学杂志,2015,6(4):251-254.

[9] Juskiewicz J,Zdunczyk Z,Zary-Sikorska E,et al. Effect of the dietary polyphenolic fraction of chicory root,peel,seed and leaf extracts on caecal fermentation and blood parameters in rats fed diets containing prebiotic fructans [J].Br J Nutr,2011(105):710-720.

[10] Xu Y,Wang L,He J,et al.Prevalence and control of diabetes in Chinese adults[J].JAMA,2013,310(9):948-959.

[11] 孙中明,肖婷,刘畅.饮食调节对糖尿病患者血糖控制的效果评价[J].吉林医学,2012,33(7):1472-1473.

[12] 方堃,周文旭,魏挺,等.2 型糖尿病患者不同治疗方式对血糖水平的影响[J].陕西医学杂志,2017,46(1):57-59.

[13] 李琳琳,杨浩,王烨,等.肠道菌群代谢产物短链脂肪酸与 2 型糖尿病的关系[J].新疆医科大学学报,2017,40(12):1517-1521.

[14] 杨远志,刘峰.益生元时代的到来[J].中国食品添加剂,2008(S1):61-67.

[15] DeFronzo RA,Ferrannini E.Insulin resistance: a multifaceted syndrome responsible for NIDDM,obesity,hypertension,dyslipidemia,and atherosclerotic cardiovascular disease[J].Diabetes Care,1991(14):173-194.

[16] Martin O,Hannah V,Matthias M,et al. Cereal fiber improves whole-body insulin sensitivity in overweight and obese women[J].Diabetes Care,2006(29):775-780.

[17] 刘凤鸣.药物和膳食纤维相结合的 2 型糖尿病综合治疗[J].常州大学学报:自然科学版,2017,29(2):29-33.

(收稿日期:2018-05-08)

综述的写作格式

综述的写作格式一般包括四部分,即前言、正文、小结、参考文献。 前言,要用简明扼要的文字说明写作的目的、必要性、有关概念的定义、综述的范围、阐述有关问题的现状和动态以及目前对主要问题争论的焦点等。正文,是综述的重点,主要包括论据和论证两个部分,正文部分根据内容的多少可分为若干个小标题分别论述。小结,是在综述正文部分作扼要的总结,作者应对各种观点进行综合评价,提出自己的看法,指出存在的问题及今后发展的方向和展望。

(C)1994-2019 China Academic Journal Electronic Publishing House. All rights reserved. http://www.cnki.net
