# Supplementary material for: A data-driven machine learning algorithm to predict the effectiveness of inulin intervention against type II diabetes
Source: Front Nutr. 2025 Jan 7;11:1520779. doi: 10.3389/fnut.2024.1520779 (PMC11747270; doi:10.3389/fnut.2024.1520779)
Supplement: Supplementary file 2 [file Data_Sheet_2.pdf]

菊粉益生元联合药物治疗老年2型糖尿病181例临床疗效观察

肖建生[1,2],张　俊[1,3],邹爱标[1,2,*],游海军[1],王青鹏[1],郭晓飞[1],姚秀芬[1],夏惠琴[4],彭淑珍[5]

(1.武汉英纽林生物科技有限公司,湖北武汉 430000;
2.清华海峡研究院医学营养研究中心,福建厦门 361006;
3.清华大学生命科学与医学研究院-英纽林肠道菌群与代谢健康研究中心,北京 100084;
4.武汉市黄陂中医院内分泌科、健康管理中心,湖北武汉 430300;
5.武汉市黄陂区人民医院内分泌科、健康管理中心,湖北武汉 430300)

摘　要:目的:观察在临床上使用菊粉益生元联合药物对60岁以上老年2型糖尿病患者的治疗效果。方法:以2017年9月~2018年9月期间在武汉市黄陂中医院和武汉市黄陂区人民医院就诊收治的181例2型糖尿病患者为研究对象,患者的年龄为60~75岁,男性57例,女性124例,平均年龄(64.88±3.55)岁。每例患者均经过2个试验周期。第一个周期遵医嘱使用药物治疗为对照组,持续用药物治疗4周;第二个周期使用药物加菊粉益生元为研究组,在对照组使用药物的基础上联合菊粉益生元治疗,药物使用剂量和方法同对照组,同时接受菊粉益生元营养强化干预,2次/d,10 g/次,餐前用温开水冲服,从第5周开始干预至第16周结束。结果:对照组使用药物治疗后血糖水平与糖化血红蛋白均显著降低($P<0.05$);总胆固醇、甘油三酯、低密度脂蛋白血脂指标显著降低($P<0.05$),分别下降了7.53%、12.14%、11.46%。研究组使用药物加菊粉益生元治疗后,血糖水平与糖化血红蛋白有极显著改善($P<0.01$);空腹血糖、餐后2 h血糖、糖化血红蛋白与对照组相比分别降低了22.1%、24.07%、18.34%;总胆固醇、甘油三酯、低密度脂蛋白血脂指标降低,分别下降了10.65%、9.87%和5.1%。对照组和研究组治疗前后的高密度脂蛋白均没有明显变化。结论:在60岁以上2型糖尿病患者的临床治疗中,使用药物联合菊粉益生元治疗效果显著,可以明显控制患者血糖、血脂水平,值得在临床应用上推广。

关键词:60岁,老年,2型糖尿病,菊粉,益生元,血糖,血脂

Clinical observation on 181 cases of senile type 2 diabetes treated by inulin prebiotic combined with drugs

XIAO Jian-sheng[1,2], ZHANG Jun[1,3], ZOU Ai-biao[1,2,*], YOU Hai-jun[1], WANG Qing-peng[1], GUO Xiao-fei[1], YAO Xiu-fen[1], XIA Hui-qin[4], PENG Shu-zhen[5]

(1.Wuhan inulin Biotechnology Co., Ltd., Wuhan 430000, China;
2.Medical Nutrition Research Center of Tsinghua Strait Research Institute, Xiamen 361006, China;
3.Institute of Life Science and Medicine, Tsinghua University-Research Center for Intestinal Flora and Metabolic Health, inulin, Beijing 100084, China;
4.Department of Endocrinology, Health Management Center, Huangpi Hospital of Traditional Chinese Medicine, Wuhan430300, China;
5.Endocrinology Department and Health Management Center of Huangpi District People's Hospital of Wuhan City, Wuhan430300, China)

Abstract: Purpose: To observe the therapeutic effect of inulin prebiotics combined with drugs on type 2 diabetic patients over 60 years old people. Methods: 181 patients with type 2 diabetes who were treated in Huangpi Hospital of traditional Chinese medicine and people's Hospital of Huangpi District of Wuhan city from September 2017 to September 2018 were selected as the study objects. The patients were 60-75 years old, 57 males and 124 females, the average age was (64.88 ± 3.55). Each patient underwent 2 trial cycles. In the first cycle, the patients were treated with drugs according to the doctor's instructions. The patients in the control group were treated with drugs for 4 weeks. In the second period, the study group was treated with drugs

作者简介:肖建生(1974-),男,硕士,工程师,研究方向:慢性病医学营养治疗,肠道菌群与代谢健康,E-mail:janson74@qq.com。
***通讯作者:**邹爱标(1970-),男,硕士,教授,研究方向:肠道菌群与代谢健康、他汀类药物研发、慢性病医学营养治疗,E-mail:zouaibiao@inuling.com。
基金项目:武汉市科技计划项目(201802040211230)。

plus inulin prebiotics.The dosage and method of the drug were the same as that of the control group.At the same time, the group received the nutritional strengthening intervention of inulin prebiotics, twice a day, 10 g a time.Before meals, they were washed with warm boiled water, from the fifth week to the end of the 16th week.Results: After treatment of the control group, the blood glucose level and glycosylated hemoglobin decreased significantly ($P < 0.05$), and total cholesterol, triglyceride, low-density lipoprotein blood lipid index were significantly reduced($P < 0.05$), 7.53%, 12.14%, 11.46% respectively.After treatment with inulin and prebiotics, the blood glucose level and glycosylated hemoglobin in the study group were significantly improved($P < 0.01$), the fasting blood glucose, 2-hour postprandial blood glucose and glycosylated hemoglobin were 22.1%, 24.07% and 18.34% lower than those in the control group, the indexes of total cholesterol, triglyceride and low-density lipoprotein were decreased by 10.65%, 9.87% and 5.1% respectively. There was no significant change of HDL in the control group and the study group before and after treatment. Conclusion: in the clinical treatment of type 2 diabetes mellitus patients over 60 years old, the effect of using drugs combined with inulin prebiotics is significant, which can significantly control the blood glucose and blood lipid level of patients, and it is worth popularizing in clinical application.

Key words: 60 years old; elderly; type 2 diabetes; inulin; prebiotics; blood glucose; blood lipid

中图分类号:TS201.3　　**文献标识码**:A

引文格式:肖建生,张俊,邹爱标,等.菊粉益生元联合药物治疗老年2型糖尿病181例临床疗效观察[J].食品工业科技,2019,40(增1):77-80.

随着生活方式和饮食结构的改变,糖尿病在全球及中国的发病率迅速上升,已经成为严重的公共卫生问题[1],然而2型糖尿病患者血糖控制的现状却仍不理想[2]。随着我国老龄化社会的来临,老年健康问题越来越受关注。伴随老年生活水平的提高,我国糖尿病患病率呈逐年上升趋势。老年糖尿病包括60岁以后才发病或60岁以前发病而延续至60岁以后的老年患者[3]。老年糖尿病的特点是症状不典型,并发症多,致残、致死率高。由于老年人饮食习惯、器官功能衰退等因素,老年糖尿病在加强药物治疗的同时更需要加强营养治疗。医学营养治疗是糖尿病患者应该长期坚持的基础性治疗措施,根据糖尿病患者的病情给予合理的营养指导能够有效的控制糖尿病患者体重,提高胰岛素对肝脏、肌肉及脂肪组织等组织器官的敏感程度,增强胰岛素作用能力,改善患者体内高血糖水平[4]。

益生元是一种能选择性刺激一种或几种细菌在宿主肠道内生长或活化,增进宿主健康而又不被宿主胃肠道消化的物质。益生元可在人体大肠内增殖大量的有益菌,同时益生元被肠道菌群发酵后可产生大量的短链脂肪酸,进而通过降低炎症因子、促进胃肠激素分泌等机制参与机体血糖调节,达到改善血糖的效果[5]。

益生元是医学营养品,使用人群广泛。有研究证实,菊粉益生元在糖尿病治疗中有较好的辅助作用,持续给予益生元营养治疗后,糖尿病患者的空腹血糖、餐后2 h血糖、糖化血红蛋白、血脂、体重均较使用前有明显改善[6-9]。

本次研究以收治的181例60岁以上2型糖尿病患者为研究对象,对药物联合菊粉益生元治疗老年2型糖尿病的效果进行了研究分析,为指导益生元联合用药治疗老年2型糖尿病提供临床依据。

1 材料与方法

1.1 材料

研究对象2017年9月~2018年9月期间在武汉市黄陂区人民医院和中医院就诊收治的181例2型糖尿病患者,年龄在60~75岁之间,其中男性57例,女性124例,平均年龄(64.88±3.55)岁;菊粉益生元武汉英纽林生物科技有限公司提供的纽畅复配益生元。

1.2 方法

以研究对象服用药物的周期(0~4周)为对照组,以服用药物加菊粉益生元的周期(5~16周)为研究组。对照组患者接受药物治疗,药物的用量和用法遵医嘱,根据个体实际情况使用,持续治疗4周。研究组患者在对照组使用药物的基础上联合菊粉益生元治疗,药物的使用方法和剂量不变,同时接受益生元营养强化干预,2次/d,10 g/次,餐前用温开水冲服,从第5周开始干预至第16周结束。采用电话随访或门诊约谈的方式对患者进行全程监督和指导。

1.3 评价指标

观察记录两组治疗前后血糖指标和血脂指标变化情况。血糖指标包括糖化血红蛋白、空腹血糖、餐后2 h血糖。血脂指标包括总胆固醇、甘油三酯、低密度脂蛋白、高密度脂蛋白。

1.4 数据处理

本次研究数据的分析处理用SAS9.4统计学软件进行统计分析,用t值进行检验,用标准差进行表示,两组数据之间的差异用P值进行判定,$P < 0.05$,具有统计学意义。

2 结果与分析

2.1 血糖指标结果

血糖指标结果如表1所示,对各指标结果分析发现,对照组使用药物治疗后,血糖水平与糖化血红蛋白均显著降低($P < 0.05$)。研究组使用药物加菊粉益生元治疗后,血糖水平与糖化血红蛋白有极显著改善($P < 0.01$)。研究组明显可以看出,空腹血糖、餐后2 h血糖、糖化血红蛋白分别降低了22.1%、24.07%、18.34%。从结果可以看出,在使用药物治疗2型糖尿病的基础上,联合使用益生元进行营养干预,可以进一步改善糖尿病患者的糖化血红蛋白

表 1　对照组和研究组治疗前后血糖水平与糖化血红蛋白指标对比

Table 1　Comparison of blood glucose level and HbA1c index between control group and study group before and after treatment

组别	对照组(n＝181)		研究组(n＝181)	
	治疗前	治疗后	治疗前	治疗后
空腹血糖(FPG,mmol/L)	9.73±3.09	8.96±2.54	8.96±2.54	6.98±1.50
餐后 2 h 血糖(2 h PG,mmol/L)	14.41±5.32	12.84±4.86	12.84±4.86	9.75±2.25
糖化血红蛋白(HbA1c,%)	7.83±1.64	7.47±1.29	7.47±1.29	6.10±0.91

表 2　对照组和研究组治疗前后血脂指标对比(mmol/L)

Table 2　Comparison of blood lipid indexes between control group and study group before and after treatment(mmol/L)

组别	对照组(n＝181)		研究组(n＝181)	
	治疗前	治疗后	治疗前	治疗后
总胆固醇(TC)	5.18±1.33	4.79±1.18	4.79±1.18	4.28±0.90
甘油三酯(TG)	1.73±1.00	1.52±0.73	1.52±0.73	1.37±0.74
低密度脂蛋白(LDL-C)	2.88±0.95	2.55±0.84	2.55±0.84	2.42±0.87
高密度脂蛋白(HDL-C)	1.34±0.38	1.33±0.41	1.33±0.41	1.33±0.39

指标和血糖水平。

2.2　血脂指标结果

血脂指标结果如表 2 所示,对各组数据分析可以看出,与试验前相比,对照组的总胆固醇、甘油三酯、低密度脂蛋白、高密度脂蛋白接分别下降了 7.53%、12.14%、11.46% 和 0.75%。研究组在经过治疗后,总胆固醇、甘油三酯和低密度脂蛋白分别下降了 10.65%、9.87% 和 5.1%,高密度脂蛋白没有变化。从结果可知,对照组和研究组治疗前后总胆固醇指标差异极显著($P<0.01$),对照组和研究组治疗前后甘油三酯指标差异显著($P<0.05$),对照组治疗前后低密度脂蛋白指标差异极显著($P<0.01$)。从整体检测数据来看,与对照组相比,药物和菊粉益生元联合使用对调控血脂有更积极的效果。

3　讨论与结论

本研究中干预对象对菊粉益生元的耐受性较好,未出现明显的不良反应,显示其具有良好的安全性,与国内外的报道一致[10-11]。

目前在临床上治疗 2 型糖尿病主要采用药物治疗,将控制患者的血糖水平作为治疗的主要目标。但在老年糖尿病患者治疗中,除了控制患者的血糖水平,还需要考虑患者的并发症。罗钰[12]认为,老年糖尿病的治疗目的是减少大血管和微血管并发症以提高生存质量和预期寿命,应根据患者情况确定个体化血糖控制目标,HbAlc 控制目标应适度放宽,生活方式干预是重要的治疗手段。蒋艳玲[13]认为,老年糖尿病患者体质较为特殊,用药禁忌较多,不但需要使用降糖的药物进行治疗,同时还需要对其合并的疾病进行综合考虑。有研究表明,老年 2 型糖尿病患者应进食能量密度高且富含膳食纤维、血糖指数低的食物[14]。因此,在老年糖尿病治疗中,在采用药物治疗的基础上进行益生元营养强化干预是可行的。

本次研究每天给患者补充 20 g 菊粉益生元,持续营养强化干预 12 周后,患者空腹血糖、餐后 2 h 血糖、糖化血红蛋白相比干预前明显降低。研究表明,益生元被肠道菌群酵解产生短链脂肪酸(如丙酸、乙酸、丁酸等),这些短链脂肪酸可以通过结合游离脂肪酸受体来下调炎症因子和上调 GLP-1 等胃肠激素分泌,参与血糖平衡调节,改善代谢性炎症和胰岛素抵抗[15]。有研究证实膳食纤维摄入量的增加能改善胰岛素的敏感性[16]。老年糖尿病患者由于器官功能开始退化,胰岛素抵抗和胰岛素敏感性都有一系列的变化。在药物治疗控制血糖水平的条件下,进行益生元营养强化干预,改善胰岛素抵抗,增加胰岛素的敏感性,改善血糖水平,是进一步改善糖尿病患者血糖的可选方案。

本次研究通过营养干预后,患者总胆固醇、甘油三酯和低密度脂蛋白与对照组相比分别下降了 10.65%、9.87% 和 5.1%,高密度脂蛋白没有变化。研究表明,丙酸影响肝脏的脂肪生成和糖异生,而菊粉还可以吸附肠道中阴离子和胆汁酸从而有效降低血脂和胆固醇[17]。相关调查显示,高血脂和糖尿病的发病率越来越高,且两种疾病会一同发展,相互作用,导致糖尿病合并高血脂的发病率也显著增高[18]。老年糖尿病患者在控制血糖水平时,更加需要控制血脂水平。在患者接受药物治疗的基础上,进行益生元营养强化干预,降低患者的血脂水平,是进一步控制血脂的可选方案。

综上所述,在 60 岁以上老年 2 型糖尿病患者的临床治疗中,使用药物联合菊粉益生元治疗效果显著,可以明显控制患者血糖水平,值得在临床应用上推广。

参考文献

[1]徐瑜,毕宇芳,王卫庆,等.中国成人糖尿病流行与控制现状—2010 年中国慢病监测暨糖尿病专题调查报告解读[J].中华内分泌代谢杂志,2014,30(3):184-186.

[2]孙飞,王丽,高彬,等.2 型糖尿病患者血糖控制情况调查分析[J].陕西医学杂志,2014,43(5):622-625.

[3]陈梅,李旭,李桂玲,等.老年糖尿病的临床特征及饮食治疗[J].中国医学创新,2009,6(13):132-133.

[4]贺婧,郭延玲.医学营养治疗联合低聚果糖干预对糖尿病患者血糖控制效果观察[J].广西医科大学学报,2018,35(3):

402-405.

[5]Liu F,Prabhakar M,Ju J,et al.Effect of inulin-type fructans on blood lipid profile and glucose level:A systematic review and meta-analysis of randomized controlled trials[J].Eur J Clin Nutr,2017,71(1):9-20.

[6]Dehghan P,Pourghassem G B,Asgharijafarabadi M.Effects of high performance inulin supplementation on glycemic status and lipid profile in women with type 2 diabetes:A randomized,placebo-controlled clinical trial[J].Health Promot Perspect,2013,3(1):55-63.

[7]Jackson K G,Taylor G R,Clohessy A M,et al.The effect of the daily intake of inulin on fastinglipid,insulin and glucose concentrations in middle-aged men and women[J].Br J Nutr,1999,82(1):23-30.

[8]Russo F,Riezzo G,Chiloiro M,et al.Metabolic effects of a diet with inulin-enriched pasta inhealthy young volunteers[J].Curr Pharm Des,2010,16(7):825-831.

[9]F Liu,M Prabhakar,J Ju,et al.Effect of inulin-type fructans on blood lipid profile and glucoselevel:A systematic review and meta-analysis of randomized controlled trials[J].Eur J Clin Nutr,2017,71(1):9-20.

[10]刘鹏举,马方,李明,等.菊粉和金玉兰对2型糖尿病患者血糖控制和血脂代谢的影响[J].协和医学杂志,2015,6(4):251-254.

[11]Juskiewicz J,Zdunczyk Z,Zary-Sikorska E,et al.Effect of the dietary polyphenolic fraction of chicory root,peel,seed and leaf extracts on caecal fermentation and blood parameters in rats fed diets containing prebiotic fructans[J].Br J Nutr,2011(105):710-720.

[12]罗钰.老年糖尿病的特点及用药分析[J].当代临床医刊,2016,29(5):2593-2593.

[13]蒋艳玲,侯惠英.老年糖尿病药物的治疗进展[J].临床医药文献电子杂志,2017,4(7):1386.

[14]Josse A R,Panahi S,Esfahani A,et al.Nutritional considerations for older adults with typ 2 diabetes[J].Nutr elder,2008,27(3-4):363-380.

[15]李琳琳,杨浩,王烨袁,等.肠道菌群代谢产物短链脂肪酸与2型糖尿病的关系[J].新疆医科大学学报,2017,40(12):1517-1521.

[16]Martin O,Hannah V,Matthias M,et al.Cereal fiber improves whole-body insulin sensitivity in overweight and obese women[J].Diabetes Care,2006(29):775-780.

[17]杨远志,刘峰.益生元时代的到来[J].中国食品添加剂,2008(S1):61-67.

[18]侯坤丽,刘颖,侯兰香.老年糖尿病合并高血脂的护理研究现状[J].糖尿病新世界,2015,16(4):157-157.

《食品工业科技》目前已被美国《化学文摘》CA、英国《食品科技文摘》FSTA、日本科学技术振兴机构数据库JST、中国科学论文统计源CSTPCD、中文核心期刊、RCCSE中国核心学术期刊、中国生物医学文献服务系统SinoMed收录期刊、中国农业核心期刊、中国科技论文在线、中国知网、万方数据、重庆维普、超星、博看等国际、国内重要检索系统或数据库收录。在2019年9月发布的《中国学术期刊影响因子年报(自然科学与工程技术·2019版)》中,期刊的影响力指数(CI值)在全国“食品科学技术”51种期刊中排名第2。
